# Examining the Obesogenic Attributes of the Family Child Care Home Environment: A Literature Review

**DOI:** 10.1155/2018/3490651

**Published:** 2018-06-10

**Authors:** Lucine Francis, Lara Shodeinde, Maureen M. Black, Jerilyn Allen

**Affiliations:** ^1^Department of Acute and Chronic Care, Johns Hopkins University School of Nursing, Baltimore, MD 21205, USA; ^2^Division of Growth and Nutrition, Department of Pediatrics, University of Maryland School of Medicine, Baltimore, MD 21201, USA; ^3^RTI International, Research Triangle Park, NC 27709, USA; ^4^Division of Internal Medicine, Johns Hopkins School of Medicine, Baltimore, MD 21205, USA; ^5^Department of Health, Behavior, and Society, Johns Hopkins School of Public Health, Baltimore, MD 21205, USA

## Abstract

Childhood obesity is a major public health concern in the US. More than a third of young children 2–5 years old are placed in nonrelative child care for the majority of the day, making the child care setting an important venue to spearhead obesity prevention. Much of the obesity research in child care has focused on center-based facilities, with emerging research on Family Child Care Homes (FCCHs)—child care operated in a home setting outside the child's home. The purpose of this review was to assess the obesogenic attributes of the FCCH environment. A search of the PubMed, Embase, CINHAL, and PsycINFO electronic databases identified 3,281 citations; 35 eligible for full-text review, and 18 articles from 17 studies in the analysis. This review found a lack of comprehensive written nutrition and physical activity policies within FCCHs, lack of FCCH providers trained in nutrition and physical activity best practices, lack of adequate equipment and space for indoor and outdoor playtime activities in FCCHs, inaccurate nutrition-related beliefs and perceptions among FCCH providers, poor nutrition-related communication with families, and poor feeding practices. Future research focusing on interventions aimed at addressing these problem areas can contribute to obesity prevention.

## 1. Introduction

Although young children 2–5 years of age in the United States (US) have experienced a decline in obesity, from 13.9% in 2004 to 8.4% in 2012, the prevalence of overweight or obesity continues to be alarmingly high, with 22.8% of young children classified as overweight or obese [1]. Young children from low-income and ethnic minority families are even more likely to be obese, compared to nonpoor and non-Hispanic White children [1, 2]. A total of 16.7% of Hispanic and 11.3% non-Hispanic Blacks are obese, compared to 3.5% non-Hispanic White and 3.4% Asian 2–5-year-olds. In 2014, data from the Centers for Disease Control and Prevention showed that 14.5% of low-income 2- to 4-year-olds who participated in the Special Supplemental Nutrition Program for Women, Infants, and Children (WIC) were obese [2].

Obesity among young children increases the likelihood of developing high blood pressure, [3] glucose intolerance [4], and poor sleep [5], all of which influence the risk for heart disease. Additionally, high hospital expenses related to complications of elevated body mass index (BMI) in young children have contributed to increasing financial burdens [6].

Much attention has been given to energy balance-related causes of obesity that are amenable to effective prevention interventions [7]. To effect change in reducing childhood obesity, a greater understanding of the environment in which children spend the majority of their time is imperative.

Parents of young children aged 2–5 years rely on early child care on a regular basis [8]. Although most children are placed in center-based child care or are cared for by relatives, nearly 2 million young children in the US are placed in Family Child Care Homes (FCCH)s, which provide nonrelative care in a home setting outside the child's home [8]. Children in child care settings eat 2-3 meals including beverages each day and have opportunities for physical activity. Young children in FCCHs are at increased risk for becoming overweight or obese, compared to children placed in center-based facilities; however, little is known about how the FCCH environment relates to childhood overweight or obesity [9–11]. Although most research related to child care and obesity has concentrated on center-based child care, research on the obesogenic attributes of the FCCH environment is emerging. We identified no reviews that have synthesized the literature on the FCCH environment. The purpose of this review was to examine the attributes of the obesogenic environment of US-based FCCHs.

## 2. Methods

### 2.1. Search Strategy and Eligibility Criteria

We searched the following electronic databases for relevant articles published in English between 2006 and 2016: MEDLINE via PubMed, EMBASE via Elsevier, CINAHL via EBSCOhost, and PsycINFO via EBSCOhost. To identify candidate studies for review, we used the keywords and controlled vocabulary terms in the following concept groups: (child care OR family child care homes OR day care OR home-based day care OR child care centers) AND (obesity OR overweight). The complete search strategy can be found in the online resource for this manuscript. We chose to review articles since 2006 coinciding with a landmark commentary on the role of child care settings in obesity prevention, highlighting the need to focus on FCCHs [12]. The final search for each database was conducted on August 8, 2016.

Studies were eligible for inclusion if they were US-based, child care studies in peer-reviewed journals that included an environmental assessment of FCCHs, and focused on FCCHs that cared for children aged 2–5 years. Nonpilot intervention studies that provided results for the assessment of the environment preintervention and studies that compared the environments of FCCHs and other types of nonrelative child care settings, including center-based facilities, were also included. The environmental assessment could have been conducted through various methods; for example, through observations, surveys, interviews, or focus groups. We excluded studies that focused on parental home settings. All search terms regarding the type of child care were used because FCCHs are described in many different ways (e.g., child care homes and home-based daycare). We also aimed to resolve any confusion of child care terms such as preschools operating out of homes. Finally, we included studies that compared FCCHs to other types of nonrelative child care settings.

### 2.2. Screening Process

The screening process occurred in two waves. In the first wave, titles, abstracts, and occasional full-text were screened to determine eligibility regarding US-based nonrelative child care studies in which the environment was assessed for children 2–5 years of age. In the second wave, titles and abstracts identified for inclusion from the first wave were further screened to identify studies that included FCCHs and assessed the environment of the FCCH setting. These studies included nonpilot intervention studies that provided results for the assessment of the FCCH environment preintervention and studies that compared the environments of FCCHs to other types of nonrelative child care settings. LF and LS independently screened the titles, abstracts, and occasional full-text. Any discordant reviews concerning eligibility were discussed and resolved. Articles identified from the second wave of screening were eligible for full-text review.

### 2.3. Data Abstraction

Articles identified for full-text review were examined for eligibility for inclusion in this review. Data from full-text articles eligible for inclusion were abstracted and included information on authorship, year of publication, the location of study, and FCCH provider level and child level demographic information (i.e., sample size, race/ethnicity, level of education, age, and BMI). Additionally, we abstracted information on the status of FCCHs based on their participation in the Child and Adult Care Food Program (CACFP), a subsidy program operated through the US Department of Agriculture (USDA), which provides reimbursements to eligible providers for the purchase of nutritious foods. Finally, we recorded the assessment findings of the FCCH environment. LF and LS examined the full-text articles for eligibility, abstracted the data, and reviewed each others' abstraction for any missing or incorrect information. We used Microsoft Excel for screening titles and abstracts and for abstraction of data. Full-text articles were read in portable document formats.

### 2.4. Classification of Studies

The articles included in the review were further classified using the Environmental Research framework for weight gain prevention (EnRG), an innovative framework grounded in behavior change-ecological theory [13]. EnRG consists of 2 frameworks. The first is the ANGELO-*AN*alysis *G*rid for *E*nvironments *L*inked to *O*besity-framework, which we used to classify the obesogenic attributes within the physical (what's available in and outside the FCCH, including education and training opportunities), sociocultural (i.e., culture around feeding practices, mealtime environment), and policy (child care policies to ensure best practices and to prevent obesity in the FCCH) environment [14]. The second is the Theory of Planned Behavior (TPB), which we used to classify articles that assessed the environment related to FCCH provider attitudes, beliefs, and perceptions [15]. These articles were organized by matching the terms and definitions used in the articles to the TPB concepts; *Attitudes* (behavioral beliefs about consequences or expected outcomes), *Subjective Norm* (normative beliefs or perception of beliefs held by most FCCH providers), *Perceived Behavioral Control* (perceived level of control to engage in best practices or perceived factors that may serve as enablers or barriers to engaging in best practices), and *Behavioral Intent* (strategies that are put in place to ensure that providers provide quality environments for the children in their care) regarding energy balance-related behaviors (EBRBs). Since knowledge is closely aligned to perceived control, provider knowledge was also classified under TPB. Demographic factors that were highlighted in the articles and included in the data analyses (i.e., neighborhood, FCCH/facility level, provider, and child level information) were classified as potential moderators. EBRBs refer to any activity that may influence children's weight in an FCCH setting. These four categories (environment, TPB concepts, potential demographic moderators, and EBRBs), which represent major components of the EnRG framework, were chosen to organize the study findings. Using this framework to help identify obesogenic attributes of the FCCH environment and EBRBs can potentially serve as a model to help guide child care researchers on how to develop tailored interventions unique to the FCCH setting.

## 3. Results

### 3.1. Results of Search

**Figure 1:**
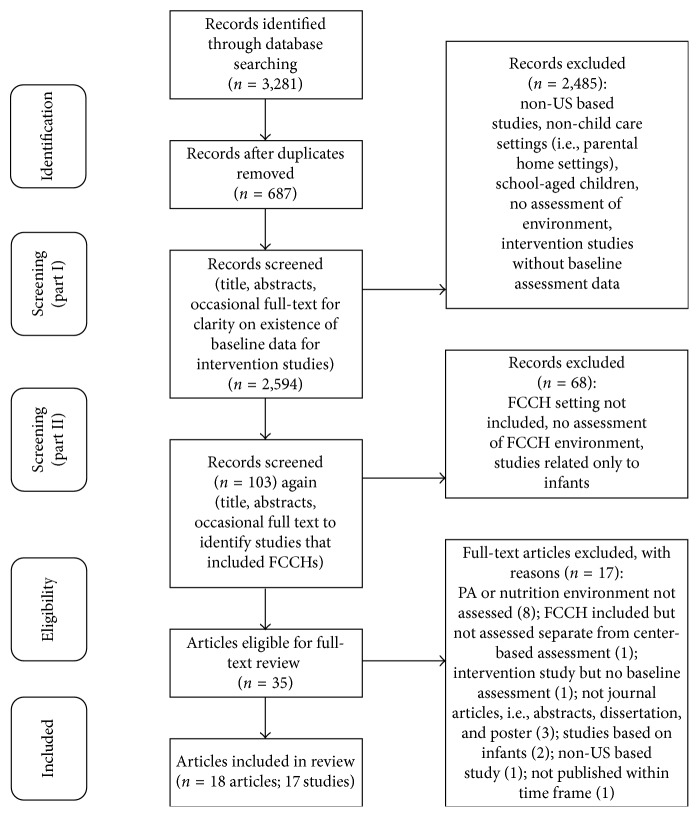
Preferred reporting items for systematic and meta-analyses diagram depicting the flow of records.

The summary of the search and screening results is shown in a flow diagram in Figure 1. A total of 3,281 records were identified from the four databases searched: 687 duplicate records were removed, and the titles of the remaining 2,594 records were screened in wave 1 for eligibility. A total of 103 records identified through wave 1 were screened for further eligibility, and 35 studies were identified for full-text review. Seventeen articles were excluded, and 18 articles were included in the review, reporting results from 17 studies.

### 3.2. Study Population

The results abstracted from the studies are summarized in Tables 1 and 2.  Table 1 displays the policy, physical, and sociocultural FCCH environment assessment results.  Table 2 displays the results from studies that assessed the FCCH environment related to providers' attitudes, beliefs, and perceptions. Per eligibility criteria, all articles included in this review involved FCCHs and assessed the environment [16–33]. There were eleven cross-sectional studies [16, 17, 19, 20, 22, 23, 25, 26, 29–32], one observational study [27], one study used accelerometers [21], and four studies used qualitative methods [18, 24, 28, 33]. Eight studies focused solely on FCCHs or FCCH providers, meaning these studies did not include other types of child care facilities [16, 18, 21, 24, 27–30]. Five studies examined both the nutrition and physical activity environment [19, 20, 26, 29, 30]. Four studies focused only on the nutrition environment [22, 23, 27, 31] while two focused solely on the physical activity environment [21, 25]. Six studies examined TPB related beliefs [16, 18, 24, 28, 32, 33]. Four of these studies used qualitative methods such as focus groups [18, 28, 33] and in-depth interviews [24]. Five studies included FCCHs participating in CACFP, [21–23, 30, 31] with 3 studies having majority (∼80%) CACFP FCCHs [21, 30, 31]. Of the studies that reported the race or ethnicity of the providers or the children, 50% (4/8) reported having majority Hispanic providers and/or children [17, 18, 20, 28]. Three studies had majority white providers [16, 21, 24], and one study involved providers who were majority African American [27]. Of the studies that reported educational level, all (7/7) reported that the majority of providers had a high school degree or GED or some college [16, 18, 21, 25, 26, 30]. Two studies reported providers' weight status; most were overweight or obese [16, 27]. Two studies reported children's weight status; most were of normal weight with 20–30% obese [21, 27].

### 3.3. Policy Environment

FCCH providers have the opportunity to have written nutrition and physical activity policies. Three studies found that few FCCH providers had comprehensive written policies on nutrition and physical activity [19, 29, 30]. Compared to center-based child care facilities, few FCCH providers had written policies regarding best practices related to beverages, the use of food as reward or punishment, and encouragement for consumption of healthy foods [19]. Trost et al. showed that fewer than 20% of FCCH providers had policies regarding best practices related to foods purchased for celebratory events [29]. Additionally, only about 25% of FCCH providers had written physical activity policies [29, 30].

### 3.4. Physical Environment

Seven studies assessed the physical environment in FCCHs [20, 22, 23, 25, 27, 29, 30]. Although more FCCH providers provided nutrition education to children, compared to center-based providers (44 versus 27%, *p*=0.01), [19, 20], few FCCH providers used books or games with nutrition themes in their delivery of nutrition education [29]. No FCCH providers reported using a dietitian to plan their menus, [22] and 44.8% of FCCH providers made water readily accessible indoors and outdoors, compared to 73.1% of centers [23]. Less than half of FCCH providers received adequate nutrition and physical activity training one or more times a year [29]. Also, the FCCH's physical activity environment was shown to be suboptimal for indoor and outdoor playtime [25, 29]. For example, Tandon et al. found that 76% FCCHs had a variety of fixed play and 86% portable play equipment when compared to center-based centers, 89% and 95%, respectively [25]. Additionally, 71% of FCCHs relied on television for part or most of the day [25]. Finally, about 22% of FCCH providers had physical activity displays such as posters, pictures, or books about physical activity [29].

### 3.5. Sociocultural Environment

Only three studies examined the sociocultural environment in the FCCH setting [17, 27, 29]. An observational study conducted in Rhode Island showed that FCCH providers frequently praised the children for trying new foods and eating healthy foods. However, in response to children's mealtime behaviors, providers used both best practices and coercive controlling practices (i.e., insistence, pressuring, and threats) when responding to children's verbal and nonverbal refusals of food, and the verbal and nonverbal acceptance of food [27]. In 85 of the interactions observed related to the providers' response for seconds, providers responded with coercive controlling practices, especially during lunch times [27]. Providers also pressured their children to “clean their plates” before offering seconds of certain foods [27]. Trost et al. showed that only 27% of FCCH providers provided family-style meals [29]. Additionally, 62.7% of FCCH providers restricted play time for misbehavior [29].

### 3.6. Theory of Planned Behavior (TBP) Concepts

There were seven articles that addressed beliefs related to knowledge, attitudes, subjective norms, perceived behavioral control, and behavioral intent [16, 18, 24, 28, 31–33]. The beliefs described in the articles were closely matched with the relevant TPB concepts. The matching of concepts was done by carefully reviewing the definitions of the concepts provided in the articles and how they were measured and matching the terms to the TPB-related concepts. Results are summarized in Table 2.

#### 3.6.1. Attitudes

Overall, two studies showed that there were poor attitudes among providers regarding parents and parents' role in fostering a healthy environment in the FCCH setting [18, 24]. For example, some providers believed that although communication with parents is important to get a better understanding of the child's well-being at home, they felt frustrated and reluctant to discuss a child's weight status with parents for fear of offending parents [18]. FCCH providers felt that the nutrition-related CACFP policies were helpful and made a difference in the health of the children attending the FCCHs [18].

#### 3.6.2. Subjective Norm

Three studies addressed subjective norms [16, 18, 28]. There were inconsistent perceptions of what was considered normal weight among FCCH providers [16, 18]. Lindsay et al. showed that despite Hispanic children being disproportionately overweight or obese, Hispanic FCCH providers reported having few children at risk for overweight or obesity or showed no concern about the weight status of the children under their care [18]. These beliefs, in turn, influenced their belief that portion sizes should be based on age and not on weight [18]. Providers,mostly white, who were presented with drawings of boys and girls of differing sizes, selected smaller sized drawings for girls as a measure for overweight, as compared to the drawing of boys [16]. These providers reported using more food restriction with girls in the FCCH, than with boys (*U*=257.5, *p*=0.10) [16]. On the topic of physical activity, most providers believed in the importance of daily physical activity in FCCHs [18]; however, the amount of time providers believed that children should engage in physical activity varied [18]. Additionally, Hispanic providers believed that 50 degrees Fahrenheit was too cold for children to play outside [28]. Although most providers perceived screen time should be limited, focus group discussions pointed to the perception among Hispanic FCCH providers that watching TV was not considered screen time [28].

#### 3.6.3. Perceived Behavioral Control

Perceived behavioral control was assessed in all six articles. Lindsay et al. showed that most providers were confident in their abilities to provide a nutritious environment for the children in their care [18]. Providers believed that they had a high level of responsibility to provide a healthy nutritional and physical activity environment and that their role was to nurture and educate the children [16, 18, 24]. Providers also perceived that they had control over what and how much children eat [18]. Providers felt that they had more influence than center-based providers on eating habits of children. However, FCCH providers also believed that both the center-based providers and FCCH providers have an equal share of influence on physical activity behavior [32]. Providers identified several enablers or barriers to engaging in nutrition and physical activity best practices. Providers believed that the high cost of food prevented the purchase of quality fresh fruits and vegetables for the children [18]. Lack of space for play was identified as a major barrier to physical activity engagement [18, 33]. Additionally, the varying needs for physical activities across ages could be challenging for providers [28, 33]. Finally, providers perceived poor parental beliefs to be an obstacle to ensuring best nutrition and physical activity practices in the FCCH [18, 28].

#### 3.6.4. Behavioral Intent

Three studies addressed providers' perceived strategies to improve the FCCH environment [18, 24, 28]. Strategies mentioned by providers included encouraging new foods, meal planning, and participating in workshops [18], problem-based solutions-oriented trainings, and programs and resources to address challenging feeding behaviors among children [28], increased reimbursement from CACFP for purchase of nutritious foods [28], improving communication with parents regarding recommended nutrition and physical activity practices [24, 28], use of dramatic play during active play time, [24] and having written, comprehensive rules inside the FCCH [24].

#### 3.6.5. Knowledge

Provider knowledge was addressed in three of the articles [24, 28, 31]. FCCH providers knew more of the rules on best nutrition practices than center-based providers in the State of Delaware (18 versus 14.7, *p* < 0.001) [31]. Providers described using their own knowledge on child development to improve what was offered to children in the FCCH [24]. Finally, providers perceived that the CACFP improved their nutrition knowledge [28]; however, this improved knowledge did not help in engaging in best feeding practices due to cultural feeding practices [28].

### 3.7. Covariates Included Analyses (Potential Demographic Moderators)

Although not directly tested for their moderation effects, this review suggests that there are certain neighborhood, FCCH/facility level, provider, and child-level characteristics that may confound relationships between the environment and EBRBs within the FCCH context.

#### 3.7.1. Neighborhood:

*Income Zone of Neighborhood.* When adjusting for the income zone of the neighborhood in which centers and FCCHs are located, indoor and outdoor physical activity and television-use practices remained significantly different between FCCHs and centers, with fewer FCCH providers providing best practices in these areas (*p* ≤ 0.05) [20]. For nutritional practices, however, the differences between FCCHs' and centers' nutritional practices were no longer significant when adjusting for the income zone of the neighborhood of the facilities (*p*=0.05) [20].

#### 3.7.2. FCCH/Facility Level

Four studies reported the number of CACFP-participating FCCHs included in the study sample [22, 23, 30, 31]. Ritchie et al. was the only study that examined the differences in environment between CACFP and non-CACFP homes [22]. CACFP and non-CACFP FCCHs were significantly more likely to serve whole milk than centers (*p* < 0.001). More non-CACFP homes served candy and sweetened beverages compared to all other types of child care settings including CACFP homes (15.8% non-CACFP homes versus 6.2% CACFP Homes, *p* < 0.001; 18.4% non-CACFP versus 7.7% CACFP homes, *p* < 0.001) [22].

#### 3.7.3. Provider Level

Hispanic providers were more likely to engage in authoritarian and controlling feeding practices. Freedman et al. found that compared to White and Asian providers, Hispanic providers (representing 76% of study population) were more likely to force children to eat what the providers perceived to be good for them (*χ* ^2^=7.25, *p* < 0.05), to insist that the children clean their plates before leaving the table, and to not allow children to eat less than they thought they should and were least likely to sit at the table and eat meals with the children (*χ* ^2^=3.04, *p* < 0.05) [17]. Hispanic providers were also three times more likely to cook foods that they knew children liked compared to Asian and White providers (*χ* ^2^=1.96, *p* < 0.001) [17]. Brann et al. demonstrated that FCCH providers (84% White) who selected smaller silhouettes for girls as overweight were more likely to have concern about the children's weight, as compared to providers who chose larger silhouettes as representing overweight (*U*=235, *p* < 0.04) [16]. Additionally, providers with higher education had fewer instances of pressuring of children to eat (*r*=−0.27, *p* < 0.01) [16]. Kim et al. demonstrated that highly trained FCCH providers were more likely to disseminate healthy nutrition information to children and obesity prevention information to parents [32].

Hispanic providers who spent their formative years in warmer climates outside the US perceived winter as a barrier to physical activity engagement more than US-born Hispanic providers [18].

#### 3.7.4. Child Level

Among 4- and 5-year-olds, overweight and obese children exhibited lower levels of moderate to vigorous physical activity and total physical activity than healthy weight 4- and 5-year-olds (*p* < 0.5). Relative to boys, girls exhibited lower levels of moderate to vigorous and total physical activity during the day (*p* < 0.5) [21].

### 3.8. Energy Balance-Related Behaviors (EBRBs)

FCCH providers reported offering more fresh fruit and vegetables than center-based child care providers (80.3% versus 51.2%, *p* < 0.001) and more frequently limiting rolls and bread compared to center-based child care providers (28.1 versus 18.6%, *p*=0.001) [20]. However, Trost et al. showed that only 41.7% of FCCH providers served lean meats more than four times per week and less than half of the providers reported serving healthy foods for celebratory events [29]. In the study by Liu et al., fewer FCCH providers reported not offering fried foods compared to center-based providers (38% versus 59%, *p*=0.001) [19].

Although FCCH providers reported following best practice recommendations for serving water at least daily and limiting sweetened beverages, 55.8% of the FCCH providers offered 100% juice 3-4 times weekly in Tandon et al. and 66% of FCCH providers in Trost et al. [26, 29]. Additionally, only 13.9% of FCCH providers offered 1% milk more than once daily [29]. Natale et al. showed that when compared to center-based child care, fewer FCCH providers provided 1% milk more than once daily (45.2 versus 55%, *p*=0.015) [20].

When compared to center-based child care, fewer FCCHs provided outside physical activity for 30 min or more three times a week (92.9% versus 96.5%, *p*=0.022) [20]. Children in FCCHs spent on average 5.8 min/hour of moderate to vigorous physical activity and 10.4 min/hour of total physical activity [21]. Although a higher portion of FCCH providers reported preschoolers engaged in 60 min of adult-led play time compared to center-based child care (33 versus 18%, *p*=0.02), only a third of FCCH providers engaged young children in an hour of playtime [19]. Seventy-eight percent of providers reported that they needed training on how to help children be physically active [19].

Nearly 65% of providers had the TV turned on every day for at least part of the day, and 55.1% of providers allowed children to watch TV or video at least once a day [29]. Natale et al. showed that more FCCH providers reported higher levels of limiting computer time than center-based child care providers (63.9 FCCH versus 51.8% centers, *p*=0.003); however, fewer FCCH providers rated excellent in limiting TV or video (39.2%, 59.5%, *p* < 0.001) [20].

## 4. Discussion

Research on obesity prevention involving FCCHs is accelerating, illustrating that a review on the obesity promoting attributes of the FCCH environment can identify priority areas for intervention development unique to the FCCH setting. With the guidance of an innovative framework, this literature review examined the obesogenic attributes of the FCCH policy, physical, and sociocultural environment. The examination of the policy environment revealed that there was lack of comprehensive written nutrition and physical activity policies within FCCHs. FCCHs are generally less regulated than center-based child care and are not mandated to have written nutrition and physical activity policies in place. Nonetheless, encouraging FCCHs to provide written nutrition and physical activity policies would provide guidance for engaging in nutrition and physical activity best practices. Children in many FCCHs have few opportunities to engage in quality physical activity due to inadequate spaces for physical activity and high television use. Many FCCH providers are also inadequately trained in nutrition and physical activity and seldom provide nutrition information to parents. There is limited research on the assessment of the sociocultural environment in FCCHs with respect to obesity and obesity prevention. In this review, there is some evidence that FCCH providers engage in controlling feeding practices and restrict physical activity as a punitive strategy for misbehavior. Since controlling and restrictive feeding styles are associated with overeating in young children, interventions aimed at reducing these obesogenic interactions are warranted [34].

The EnRG framework postulates that the environment can have an effect on EBRB through the mediating role of certain cognitive factors; the TPB concepts. In this review, six articles explored FCCH providers' attitudes, normative beliefs, and control beliefs as they pertain to the FCCH environment and obesity. Although mediation was not examined, the evidence suggests that providers' attitudes and beliefs influence their feeding and physical activity practices as well as family communication practices. Further understanding of these concepts as they relate to the FCCH environment would be instrumental in developing training strategies that can eliminate misconceptions and inappropriate beliefs about nutrition and physical activity practices and enhance self-efficacy, which would help with better communication with families concerning children's eating behaviors. Partnering with families is likely to be effective since families engage with child care providers daily and often share information on the child's daily activities.

We abstracted information on the covariates that were collected and/or included in the analyses. Although the covariates were not tested for moderation, the evidence suggests that many of the covariates may function as potential demographic moderators that should be tested in future research. Within the EnRG framework, demographic moderators may confound relations between the mediated environment and EBRBs or relations between the unmediated and automatic-lack of awareness or control-environment and EBRBs.

Although most studies did not examine neighborhood differences, one study adjusted for neighborhood characteristics in comparing FCCH with child care centers [20]. The finding that nutrition-related FCCH/center differences were eliminated after neighborhood adjustment suggests that FCCH and center nutritional practices may be related to neighborhood conditions, such as food availability. In contrast, that finding that neighborhood adjustment did not alter that finding that FCCHs had worse physical activity practices than center-based child care suggests that FCCH/center differences may be more closely related to physical activity resource and practice differences within the sites, rather than the neighborhoods.

Only one study examined the differences in the food environment by CACFP status [22]. The finding that non-CACFP homes served candy and sweetened beverages more often than CACFP homes is consistent with a study that showed that compared to non-CACFP providers, more CACFP providers engaged in best nutrition practices [35]. Since thirty percent of US children are enrolled in CACFP-participating FCCHs, more research is needed to examine the impact that CACFP is making on the food environment and feeding practices in FCCHs especially since the introduction of new CACFP guidelines on October 1, 2017, to increase the consumption of fruits and vegetables and reduce grain-based deserts, such as donuts and pastries [36].

On the provider and child-level, FCCH interventions are needed to address Hispanic providers' propensity to use controlling and coercive feeding practices, along with their inaccurate beliefs concerning physical activity and TV use. Provider nutrition training was effective in empowering providers to disseminate healthy nutrition-related information to parents and children [32]. Strategies to increase funding and availability for nutrition training programs for FCCH providers, through CACFP or independent of CACFP, could be instrumental in helping to curb obesity among young children.

Finally, FCCH providers engage in obesogenic EBRBs, providing fried foods, high-fat milk, sweetened beverages, and having limited opportunties for play both indoors and outdoors coupled with high TV use. A mixed-methods approach can help rearchers to understand FCCH providers' knowledge in nutrition and physical activity best practices and barriers to providing an optimal environment for the children in their care. Developing social marketing campaigns tailored for FCCH providers can be an effective approach to influence behavior change.

### 4.1. Limitations and Strengths

There are several limitations to this review that can affect the generalizability of the findings. The few studies that examined the FCCH environment were limited to only several states, with no national representation. We limited the search to published literature, not grey literature, meaning that unpublished studies or published studies in a noncommercial form were excluded. Since most studies did not report the licensure status and the size of the FCCHs, we were unable to make inferences concerning the role of licensure and size on EBRBs. Half of the studies that reported race or ethnicity involved majority Hispanic providers and only one study had majority African American providers. Of the studies that examined the policy, physical, and sociocultural environment, only one study relied on observations to assess the environment [27]. Most studies relied on self-report cross-sectional survey data, potentially introducing biases that could be minimized by objectively observing the FCCH environment. Due to the studies' heterogeneity in design, we were unable to evaluate the quality of the studies using one tool. Finally, none of the studies examined the food environment in the neighborhoods outside of the FCCH. Expanding the child care research network to include FCCHs across the US, striving for equal representation in races/ethnicities of FCCH providers and children in their care, addressing neighborhood characteristics, including systematic and observational methods that examine policies and practices, and examining FCCHs over time, would improve generalizability in the process of defining the FCCH environment in the US.

There are also multiple strengths to the review. FCCHs are a primary resource for many families and are an ideal venue for the implementation of childhood obesity prevention efforts. With strong theoretical guidance, we are able to show that making changes in the policy, physical, and sociocultural environment of FCCHs can provide optimal environments for young children. Enhancing nutrition training for providers and promoting healthy mealtime interactions may improve children's dietary environment. Lastly, we show the need for more studies to understand the impact CACFP has on the food environment of FCCHs.

## 5. Conclusion

In conclusion, this review of the obesogenic attributes of the Family Child Care Home highlights the priority areas in which to intervene within the FCCH environment. Interventions addressing child care policies and practices regarding what food is served, how food is served, opportunities provided to young children for physical activity, and the quality of space available within FCCHs, are essential. With better opportunities for FCCH providers to be trained in childhood obesity prevention and in best practices in nutrition and physical activity, providers can be proactive in implementing written nutrition and physical activity policies. FCCH providers would benefit from innovative strategies to implement physical activity and minimize screen time, given space limitations. Finally, addressing misconceptions and inappropriate attitudes and beliefs related to food and physical activity can benefit the health of the FCCH environment. Ensuring that all child care options for young children, including FCCHs, including the policies, practices, and resources to help children build healthy nutrition and physical activity habits, can be instrumental in preventing overweight and obesity.

## Figures and Tables

**Table 1:** The policy, physical, and sociocultural environment of family child care homes.

Citation/year/state/method	Sample description	Data source/measures	Policy environment	Physical environment	Sociocultural environment	EBRBs	Covariates in analyses (potential demographic moderators)
	Nutrition						
Freedman and Alvarez [17], *Journal of the American Dietetic Association*/2010/CA/Pre-post test; cross-sectional analyses of pre-test phase	*N*=54% (39) FCCHs; 46% center-based ^a^ **Provider** *Race/ethnicity*: 76% of FCCHs Hispanic *Age*: 18 years+	Modified SCFQ and HCFSQ			*FCCH* *>* *Centers* cooking foods children liked (63 versus 39%, ns) allowed children to eat less than they think they should (47 versus 29%, ns) Rarely/never allowed children to eat more than they thought they should (55 versus 27%, ns)		**Ethnicity** *Hispanics* less likely to eat meals with children (diff: 0.24, *χ* ^2^=3.04; *p* < 0.05); more likely to force children to eat what is “good for them” (*χ* ^2^=7.25, *p* < 0.05). 3x more likely to cook foods they knew children liked (*χ* ^2^=1.96, *p* < 0.001) 50% insisted children finish food before leaving the table

Liu et al. [19], *Maternal and Child Health Journal*/2016/OH/cross-sectional survey	*N*=185 child care settings; 44% FCCHs; 56% centers	Modified NAPSACC and EPAO	*FCCHs* *<* *Centers re policies* *Beverages* only milk, water, and 100% fruit juice served; (47 versus 77%, *p*=0.001) <6 oz of 100% fruit juice/day served to ≥age 12 months (22 versus 43%, *p*=0.003) skim, 1 or 2% milk served >age 2 years (28 versus 50%, *p*=0.003) No eating/drinking outside foods (12 versus 31%, *p*=0.003) *Use of food as punishment/reward* cannot withhold/delay food/drinks as punishment; (44 versus 83%, *p*=0.001) cannot give food/drinks as a reward or an incentive (30 versus 48%, *p*=0.01) *Authoritarian/controlling feeding interactions* No forcing children to eat certain foods or quantities; (33 versus 55%, *p*=0.004) allowing children to decide how much to eat (25 versus 38%, *p*=0.05) Encouraged (not forced) to eat/taste food (30 versus 45%, *p*=0.03)	*FCCHs* *>* *Centers* teach about food ≥1x/mos. (44 versus 27%, *p*=0.01)		*FCCHs* *<* *Centers* not offering fried foods (38% versus 59%, *p*=0.001)	
Natale et al. [20], *Early Childhood Education Journal*/2014/FL/cross-sectional survey of baseline data	*N*=298 FCCHs; 842 center-based **Provider/home** ^**b,c**^ *SES*: Facilities' zip code = 18.1% > 40% of household income <25K **Children** *Race/ethnicity* Enrollment 8.3% FCCHs predominantly black 45.8% FCCHs predominantly Hispanic	Modified HSFFQ		*FCCHs* *>* *Centers* provided more lessons with a basis in health and nutrition/week (*p*=0.036)		*FCCHs* *<* *Centers* provided 1% milk >1x/day (45.2 versus 55%, *p*=0.015) *FCCHs* *>* *Centers* provided more fresh fruit (*p*=0.001), limited servings of rolls/bread (28.1 versus 18.6%, *p*=0.001)	**Income zone of facility** Adjusting for income zone: (ns associations between facility type and all other nutritional/dietary outcomes (*p* > 0.05)

Ritchie et al. [22], *Childhood Obesity*/2012/Multi-state/cross-sectional survey	*N*=65 Head start centers; 68 preschools; 104 CACFP centers; 88 non-CACFP centers; 65 CACFP FCCHs; 38 non-CACFP FCCHs	Adapted NAPSACC		*FCCHs* *<* *state preschools, centers* Used dietitian in menu planning (0% versus 19.1%, 4.2%, *p* < 0.001)			**CACFP status** *CACFP and non-CACFP FCCHs* *>* *Centers* served whole milk (*p* < 0.001). *non-CACFP FCCHs* *>* *CACFP FCCHs* served candy day before survey (15.8% versus 6.2, *p* < 0.001) served sweetened drinks day before the survey (18.4% versus 7.7%, *p* < 0.001)

Ritchie et al. [23], *Preventing Chronic Disease*/2015/CA/cross-sectional survey in 2008 and 2012	*N*=429 child care sites (2008); 435 child care sites (2012); 65 CACFP homes; 38 non-CACFP homes	Adapted NAPSACC		*FCCHs* *<* *Centers* made water easily available to children for self-service indoors and outside (44.8% versus 73.1%, *p* < 0.001) provided tap water (*p*=0.01)			

Tandon et al. [26], *Journal of Nutrition Education and Behavior*/2012/FL, MA, MI, WA/cross-sectional surveys	*N*=94 FCCHs; 74 center-based **Provider** *Level of education*: 28% HS grad, 21% some college, 51% 2 or 4 year college	NAPSACC				FCCHs + Centers = follow best practice recommendations for serving water at least daily + sugar sweetened beverages 66% FCCHs and centers offered 100% juice 3-4 times weekly	
Tovar et al. [27], *Appetite*/2016/RI/Observational study	*N*=48 FCCHs; 214 observed meals and snack times; 227 child-provider interactions captured **Provider** *Race/ethnicity*: 75% African-American 19% white *Gender*: 100% female *Level of education*: 57% HS or associates, 40% Bachelors *BMI*: 77% obese 18% overweight **Children** *BMI* 67% normal weight 13% overweight 20% obese	Modified EPAO			**Only plated meals served** **Response to verbal refuses of food** 55% best feeding practices in response to verbal refuses 45% coercive controlling practices **Response to nonverbal refuses of food** both best practices and coercive controlling practices equally **Response to verbal and non-verbal acceptance of food** reacted to food acceptance with autonomy supportive practices > coercive controlling practices (43 versus 5 interactions) **Response for seconds** 85% responded with coercive controlling practices, esp. during lunch pressured children to clean their plates first to get seconds of certain foods some simply complied/offered bribes Being “all done” responded equally with coercive and best practices Pressuring children to eat more frequently observed **Attempts for praise or attention** frequently praised for trying new foods and eating certain foods		

Trost et al. [30], *American Journal of Preventive Medicine*/2011/KS/cross-sectional survey	*N*=297 FCCHs; 85.3% CACFP participation **Provider** *Level of education*: 40.8% HS diploma or GED, 42.9% some college or Associate's, 14.3% Bachelor degree	^d^NAPSACC *M*(sd)	Nutrition policy 2.41 ± 0.5	Menus and variety 2.50 ± 0.6 Nutrition education 2.60 ± 0.7		**Foods served** Fruits and vegetables 3.20 ± 0.4 Fried foods and high-fat meats 3.10 ± 0.3 Beverages 2.90 ± 0.5 Meals and snacks 3.70 ± 0.3 Foods outside of regular meals and snacks 2.00 ± 0.7 Supporting healthy eating 3.00 ± 0.5	
Trost et al. [29], *American Journal of Preventive Medicine*/2009/KS/cross-sectional survey	*N*=297 FCCHs	NAPSACC	Written guidelines concerning type of foods brought for celebrations 18.6% (95% CI: 13.7, 23.4) comprehensive written policy on nutrition and food services 53.7% (95% CI: 47.6, 59.7)	Received nutrition training ≥1x/yr 47.5% (95% CI: 41.2, 53.8) offered nutrition education for children 46.9% (95% CI: 40.6, 53.2) offered nutrition education to parents 45.3% (95% CI: 39.1, 51.5)	Family-style meals 23% (95% CI: 17.7, 28.4)	Served lean meats >4x/wk 41.7% (95% CI: 35.4, 48) served 100% fruit juice >1x/day 55.8% (95% CI: 49.6, 62) Served 1% milk 13.9% (95% CI 9.7, 18.1) Infrequent use of healthy foods for celebrations 43.9% (95% CI: 37.6, 50.2)	

	Physical activity						
Liu et al. [19], *Maternal and Child Health Journal*/2016/OH/cross-sectional survey	*N*=44% FCCHs; 56% centers	Modified NAPSACC and EPAO				*FCCHs* *>* *Centers* preschoolers engaged in 60 min of adult-led physical activity/day (33 versus 18%, *p*=0.02) required training on how to help children be physically active (78 versus 56%, *p*=0.002).	

Natale et al. [20], *Early Childhood Education Journal*/2014/FL/cross-sectional survey of baseline data	*N*=298 FCCHs; 842 center-based child care **Provider/home** ^b,c^ *SES*: Facilities' zip code = 18.1% > 40% of household income <25K **Children** *Race/ethnicity*: Enrollment: 8.3% FCCHs predominantly black 45.8% FCCHs predominantly Hispanic	^e^PAFQ				*FCCHs* *<* *Centers* provided outside PA for 30 min + /3x/wk. (92.9% versus 96.5%, *p*=0.022) rated excellent in amount of limiting TV/video (39.2%, 59.5 %, *p* < 0.001) *FCCHs* *>* *Centers* reported higher levels of limiting computer time (63.9 versus 51.8%, *p*=0.003)	**Income zone of Facility** Adjusting for income zone: sig. Differences remained b/t indoor physical activity, outdoor physical activity, and television-use practices (*p* ≤ 0.05)
Rice and Trost [21], *Journal of Nutrition Education and Behavior*/2014/OR/accelerometer readings	*N*=47 FCCHs, 114 children (60 boys, 54 girls), 70% CACFP **Provider** *Age*: 2% less than 30, 44% 30–39, 54% greater than 40 *Provider race* 90% white, *Mean yrs. of operation*: 10 (IQR 5–15) *Level of education* 66% HS diploma or GED, 20% some college or associate degree, 15% Bachelor's degree **Children** *Avg. BMI* 16.8 ± 202% *overweight or obese*: 29%	ActiGraph GT1M accelerometer				Avg. participation in MVPA and total PA 5.8 ± 3.2 and 10.4 ± 4.4 min/h, respectively	**BMI** overweight and obese 4-5 yr olds < healthy 4-5 yr olds MVPA and TPA (*p* < 0.5) **Gender** Girls < boys exhibited MVPA and TPA (*p* < 0.5)

Tandon et al. [26], *Journal of Nutrition Education and Behavior*/2012/FL, MA, MI, WA/cross-sectional surveys	*N*=94 FCCHs; 74 center-based **Provider** *Level of education*: 28% HS grad, 21% some college, 51% 2 or 4 year college	NAPSACC		*FCCHs* *<* *Centers* variety of fixed-play equipment (76 versus 89%, *χ* ^2^=5.3, *p*=0.02) variety of portable play equipment (86 versus 95%, *χ* ^2^=4.4, *p*=0.04) rarely or never showed TV (29 versus 68% *χ* ^2^=25, *p* < 0.001)		50% of preschoolers in FCCHs <1 hr/day outdoor play time	
Tandon et al. [25], *Academic Pediatrics*/2012/cross-sectional analyses on longitudinal data	*N* = Overall (1900); Nonrelative in Child's home (*n*=150); Nonrelative in another home (*n*=550) Provider (*nonrelative in child's home, nonrelative in another home*) **Race**: 85%, 82% white, 6%, 13% Black, 26%, 14% Hispanic **Level of education** 34, 37% HS or less 35, 45% some college 26, 14% college graduate 6, 4% graduate degree	ECLS-B				50% of home-based providers take the child outside to walk or play ≥1x/day Nonrelatives in home-based > relatives in homes Increased odds of going outside daily for children (OR 1.5, 95% CI 1.36–1.64). Nonrelative care in another home ≠ relative care Odds of outdoor play did not differ	

Trost et al. [30], *American Journal of Preventive Medicine*/2011/KS/cross-sectional survey	*N*=297 FCCHs: 85.3% CACFP participation **Provider** *Level of education*: 40.8% HS diploma or GED, 42.9% some college or Associate's, 14.3% bachelor degree	NAPSACC *M*(sd)	PA policy 1.6 ± 1.2	Play environment 3.10 ± 0.6 Physical activity education 2.2 ± 0.9	Supporting physical activity 2.40 ± 0.7	Active play and inactive time 3.20 ± 0.4 TV use and TV viewing 2.90 ± 0.8	

Trost et al. [29], *American Journal of Preventive Medicine*/2009/KS/cross-sectional survey	*N*=297 FCCH providers	NAPSACC	Existence of comprehensive written policy on PA 24.9% (95% CI: 19.5, 30.3)	Suitable space indoors when weather is bad 17.6% (95% CI: 12.8, 22.3) Displayed posters, pictures, or books about PA 21.9% (95% CI: 16.5, 27.2) Received training on PA ≥1x/per year 46.1% (95% CI: 39.8, 52.3): Provided PA education to parents 30.2% (95% CI: 24.3, 36)	Restricted active play time for misbehavior 62.7% (95% CI: 56.6, 68.7)	TV turned on every day for at least part of the day 64.6% (95% CI: 58.7, 70.5) 55.1% (95% CI: 48.7, 61.4) allowed children to watch TV/videos ≥1x/day	

^a^Included results regarding ethnicity since a great percentage of homes were Hispanic; ^b^significantly different from center based; ^c^FCCHs more likely to care for children enrolled in federal subsidy programs; ^d^Scoring guide: 1 = marginally meeting child care standards, 2 = meeting child care standards, 3 = exceeding child care standards, and 4 = far exceeding child care standards and using best practice; ^e^developed based on physical activity standards from Caring for Our Children National Health and Safety Performance Standards; SCFQ = Stanford Child Feeding Questionnaire; HCFSQ = Hughes Caregiver Feeding Styles Questionnaire; EPAO = Environment and Policy Assessment and Observation; HSFFQ = Harvard Service Food Frequency Questionnaire; PAFQ = Physical Activity Frequency Questionnaire; ECLS-B = Early Childhood Longitudinal Study-Birth Cohort; FCCHs = Family Child Care Homes; NAPSACC = Nutrition and Physical Activity Self-Assessment for Child Care; CACFP = Child and Adult Care Food Program; PA = physical activity; MVPA = moderate to vigorous physical activity; TPA = total physical activity; M *=* mean; SD = standard deviation; ns = not significant; mos. = months.

**Table 2:** TPB Concepts_Attitudes, subjective norm, perceived behavioral control, intent and practices + knowledge.

Citation/year/state/method	Sample description	Study concepts (*related TPB concepts*) and article's definition	Results on TPB-related concepts	Association between TPB concepts and child care practices	Covariates in analyses (potential demographic moderators)
Brann [16], *Journal of Pediatric Health Care*/2010/Onondaga County, central NY/Cross-sectional survey	*N*=123 FCCH providers **Provider** ^*a*^ *Race*: *84% white, 11% black, 2% hispanic, 0.8% native American* *Age*: 45% 20–40, 50% 41–60, 5% greater than 60 years *Education level*: 30% college graduate or above, 66% HS graduate or some college, 4% some HS *Avg. BMI*: 27 ± 7.7 **Children** *Avg. age*: 4.5 years ± 1.5 *Avg. household Income*: 42% < 40K, 52% 40–80K, 6% > 80K	**Perceptions of childhood overweight** (*subjective norm*): perception of what is considered overweight by identifying drawings of boys and girls ranging from very thin to very heavy that lie in a gradient from thin to heavy **Perceived responsibility in child feeding** (*perceived behavioral control*)	**Perception of childhood overweight** (*subjective norm*) Most providers chose a figure representing an above average-sized boy and girl as a cut off point for overweight **Perceived responsibility in child feeding** (*perceived behavioral control*) High level of responsibility for feeding and monitoring	**Perception of childhood overweight** (*subjective norm*) Providers who selected smaller silhouettes for girls as a measure for overweight reported using more food restriction on girls (*U*=257.5, *p*=0.10).	**Level of Education** Providers with a higher level of education were correlated with less pressuring of children to eat more food (*r*=−0.27, *p* < 0.01) **Concerned about child weight** Relationship exist between concern about weight and restriction of unhealthy foods (*r*=0.27, *p* < 0.01) Providers who selected smaller silhouettes for girls were more likely to have more concern about the child's weight (*U*=235, *p* < 0.04)

Kim et al. [32], *Maternal and Child Health Journal*/2012/East Central Illinois/Cross-sectional analysis	*N*=88 FCCH providers; 94 center-based providers	**Perceptions** (*perceived behavioral control*) providers' perceptions of the level of influence on children's healthy behaviors and weight status	**Providers' perceptions of the level of influence on children's healthy behaviors and weight status** (*perceived behavioral control*) Both the family and center-based providers felt that the home environment had more influence on healthy eating/PA habits and weight status of the children (*paired t tests, all significant p values*) FCCHs > centers ranked influence on health behaviors and weight status with the exception of PA. FCCH providers felt that home and center-based facilities shared similar influence on physical activity of the children.		**Level of training** **results** >55% of FCCH providers = obesity prevention training within the past year, which is a marked difference 30% of center-based providers (*χ* ^2^, *p*=0.0005). Highly trained FCCH providers more likely to receive nutrition and PA training (*χ* ^2^, *p*=0.0009, 0.0024 respectively) Highly trained FCCH providers more likely to disseminate healthy nutrition and PA information to children (PA and obesity prevention information to parents (*all significantpvalues*)
Lindsay et al. [18], *Journal of Obesity*/2015/MA/Focus groups	*N*=44 Latino FCCH providers; 4 focus groups **Provider** *Ethnicity* 100% Latino *Education level* −1/3 HS graduate or GED, 40% some college *Years of experience* 93% up to 25 years of child care experience	**Provider's perceptions, attitudes and practices related to nutrition and physical activity** (*attitudes*) perceptions of the CACFP and EEC (*attitudes*) attitudes related to communication with parents (*subjective norm*) perception of child weight status of kids in care of providers (*subjective norm*) beliefs about physical activity and sedentary behaviors (*perceived behavioral control*) provider control on what and how much children eat (*perceived behavioral control*) perceived barriers to PA and healthy eating (*perceived behavioral control*) provider's belief related to their role (*perceived behavioral control*) perceived barriers to provision of healthy foods (*Behavioral Intent*) Strategies to incorporate nutritious foods	**Attitudes towards CACFP and EEC** (*attitudes*) CACFP policies helpful and made a difference in the health of children. **Attitudes related to communication with parents** (*attitudes*) communication with parents important and critical in understanding child's well-being at home did not feel comfortable discussing children's weight status with parents. **Perceptions of child weight status** (*subjective norm*) Few providers reported having some children at risk for overweight or obesity Most reported that they did not have major concerns about weight status of children currently under their care. **Beliefs about PA and sedentary behavior** (*subjective norm*) Most believed the importance for children to engage in PA throughout the day. However, the amount of time providers believed children should engage in PA varied (from 30 minutes to 2 hours) **Foods served and portion sizes** (*perceived behavioral control*) perceive parents to be a barrier to healthy eating in FCCH **Beliefs related to child feeding** (*perceived behavioral control*) Perceived role is to nurture and educate children Most feel confident in the abilities to serve healthy foods **Perception on need to control feeding** (*perceived behavioral control*) Felt need to control what and how much children eat **Perceived barriers to PA** (*perceived behavioral control*) Most believed lack of space and cold whether to be major obstacles for PA opportunities **Perceived barriers to provision of healthy foods** (*perceived behavioral control*) Perceived high cost of fresh fruits and vegetables does not enable them to purchase and provide these foods. Perceived that the CACFP does not pay enough for purchase of fresh fruits and vegetables **Strategies to incorporate nutrition foods** (*behavioral intent*) Encouraging new foods Meal planning Participating in workshops	**Attitudes related to communication with parents and communicating weight concerns to parents** (*attitudes*) Providers who reported being uncomfortable and reluctant to discusschild's weight felt that parents can be very sensitive to other people's perceptions of their children, and because of that they preferred not to talk about it with parents. **Foods served and portion sizes** (*subjective norm*) Providers report serving foods aligned with recommendations from USDA Many providers base portion sizes on age of child **Perceptions of child weight status and determining portion sizes** (*subjective norm*) Few providers reported having some children at risk for overweight or obesity and that this influenced their feeding practices, especially in determining portion sizes	**Providers' place of birth** Providers who had formative years outside of US, in warmer climates, perceived winter as a barrier to PA more than US-born providers
Rosenthal et al. [24], *Journal of Nutrition Education and Behavior*/2013/CT/in-depth interviews	*N*=17 FCCH providers **Provider** *Race/ethnicity* 29% African American, 53% white, 24% Latina *Mean age* 43 yrs (31–54) *Mean yrs working in child care* 13 (5–32) *Household income* less than 50K (47%), 50–75K (29%), 75–100K (18%), more than 100K (6%)	**Attitudes towards parents** (*attitudes*) **Perceived role in obesity prevention** (*perceived behavioral control*) **Strategies used to implement best practices in nutrition and PA** (*behavioral intent*)	**Attitudes towards parents** (*attitudes*) expressed both empathy and frustration with parents. **Perceived role in obesity Prevention** (*perceived behavioral control*) perceived they had a personal responsibility in obesity prevention. Discussed the importance of their role in sharing healthy foods with parents. acknowledged the supportive role of food guidelines, unannounced inspections from the government sponsored food program, and the peer group.	**Strategies used to implement best practices in nutrition and PA** (*behavioral intent*) described how, at the first meeting with parents, they try to be clear with parents about food guidelines. Some have written rules about food guidelines and all described having a conversation with families about food guidelines. described using their own knowledge of child development to improve nutritional intake and incorporating dramatic play to facilitate times of high PA described how they incorporate another aspect of child development, socialization, to improve a child's nutrition. described how they use dramatic play to facilitate PA described sharing with parents both the actual food and the techniques they use to encourage children to eat nutritiously did not pressure kids to eat but were still concerned so helped the child to eat	
Tovar et al. [28], *Childhood Obesity*/2015/RI/Focus groups	*N*=30 FCCH providers; 4 focus groups **Provider** *Race/ethnicity* 100% female, Hispanic (predominantly Dominican, 77%), and Spanish speaking *Level of education* 50% = some college+ *Mean age* 50 years	**Perceptions on use of TV** (*subjective norm*) **Perceptions and beliefs** regarding which factors influence children's PA, screen time and dietary behaviors (*perceived behavioral control*) **Perceived strategies to improve the nutrition and PA environment of FCCHs** (*behavioral intention*)	**Provider perceptions on screen time behaviors** (*subjective norm*) Screen time should be limited and rules should be in place to stop parents from leaving children at FCCH with electronic devices Perceived watching educational programs such as Dora the Explorer learning and not screen time Use of TV for food prep Others felt that watching TV did not benefit child **Provider's perceptions on how preschool-aged children can be physically active** (*perceived behavioral control*) Perceived that children have many opportunities to engage in PA in the home Perceived that children are more active when outside Perceived that there are opportunities indoor but needs to be scheduled into the provider's day **Influences on what and how providers feed or offer PA opportunities for preschool-aged children** (*perceived behavioral control*) Perceived responsibility to provide children with nutritious foods Perceived need to abide by program regulations, though some deem regulations as contributing to added stress Culture influenced foods served Poor parental behavior influences the childcare environment Providers perceive parents' poor beliefs regarding PA to be a major barrier to PA in the FCCH. Providers perceive children's varying preferences to be a barrier to group PA Fear of children getting hurt in home limits PA in home Winter weather Provider perceived 50F to be too cold to take children outside **Perceived strategies to improve the nutrition and PA environment of FCCHs** (*behavioral intent*) More problem-based solutions oriented trainings, programs and resources Increased reimbursement for purchase of fruits and vegetables Improve communication with parents regarding proper nutrition and PA practices	**Training and feeding practices** Often disconnect between providers belief on the importance of healthy foods and what they actually serve Perceived the CACFP program to help enhance knowledge on nutrition foods, yet some still do not follow nutrition guidelines of the food program due to cultural feeding practices Providers relied on child's age and physical stature to determine portion size instead of relying on age-appropriate guidelines for portion sizes Due to training, providers appreciated the importance of not force feeding and being a role model during feeding mealtimes	
Vinci et al. [33], *Journal of Obesity*/2016/FL/Focus groups	*N*=27 FCCHs (75.9% of sample of child care providers)	**Subjective beliefs of what is needed to ensure PA in homes** (*perceived behavioral control*)	**Subjective beliefs of what is needed to ensure PA in homes** (*perceived behavioral control*) additional specific factors that were not raised by center staff including the need for activities that can be adapted for a wide range of ages. Home providers cautioned against providing PA that required extensive space or equipment, since space is limited in FCCHs.		

Van Stan et al. [31], *Childhood Obesity*/2013/DE/survey	*N*=62% FCCHs; 5% center owner; 84% CACFP	**Knowledge** of nutrition and PA rules		***FCCHs*** ***>*** ***Center staff*** Knowledge of DE's nutrition and PA rules (14.7 versus 18 out of 26, *p* < 0.001)	

^a^Missing data for five providers; FCCH = Family Child Care Homes; EEC = Early Education and Care; BMI = body mass index; PA = physical activity; MVPA = moderate to vigorous physical activity; TPA = total physical activity; *U* = Mann–Whitney test.

## Supplementary Materials

Supplementary Materials Complete search strategy for this review. Final search conducted on August 8, 2016.
