# Supplementary material for: Examining the Obesogenic Attributes of the Family Child Care Home Environment: A Literature Review
Source: J Obes. 2018 Jun 10;2018:3490651. doi: 10.1155/2018/3490651 (PMC6015672; doi:10.1155/2018/3490651)
Supplement: Supplementary Materials — Complete search strategy for this review. Final search conducted on August 8, 2016. [file 3490651.f1.docx]

# Search Summary

3281 Total Citations

2600 Citations after Duplicates Removed (1681 duplicates)

# Search Strategies

## PubMed

((("Child Day Care Centers"[Mesh] OR "Schools, Nursery"[Mesh] OR "child care center" [tiab] OR "nursery school" [tiab] OR "nursery schools" [tiab] OR "child care" [tiab] OR "home-based day care" [tiab] OR "family child care home" [tiab] OR "family child care homes" [tiab] OR "child care centers" [tiab] OR "daycare" [tiab]))) AND (("Obesity"[Mesh] OR "Overweight"[Mesh] OR adipose tissue [mh] OR abdominal fat [mh] OR weight gain [mh] OR weight loss [mh] OR body mass index [mh] OR skinfold thickness [mh] OR waist-hip ratio [mh] OR overweight* [tw] OR "fat overload syndrome" [tw] OR "fat overload syndromes" [tw] OR overeat*[tw] OR overfeed* [tw] OR adipos* OR obes* OR "body mass index" OR bmi [tw] OR "waist-hip ratio" OR "skinfold thickness" OR "abdominal fat"))

Limited 1/2006 to present

Searched 08082016

452 citations

## **Embase**

| Search Queries  \| **No.** \| **Query** \| **Results** \| **Date** \| \| --- \| --- \| --- \| --- \| \| #4 \| ('obesity'/exp OR 'childhood obesity'/exp OR 'body mass'/exp OR 'waist hip ratio'/exp OR 'overnutrition'/exp OR overweight*:ab,ti OR 'fat overload syndrome':ab,ti OR 'fat overload syndromes':ab,ti OR overeat*:ab,ti OR overfeed*:ab,ti OR adipos*:ab,ti OR obes*:ab,ti OR 'body mass index' OR bmi:ab,ti OR 'waist-hip ratio' OR 'skinfold thickness' OR 'abdominal fat') AND ('child care'/exp OR 'day care'/exp OR 'nursery school'/exp OR 'child care center':ab,ti OR 'nursery school':ab,ti OR 'nursery schools':ab,ti OR 'child care':ab,ti OR 'home-based day care':ab,ti OR 'family child care home':ab,ti OR 'family child care homes':ab,ti OR 'child care centers':ab,ti OR 'daycare':ab,ti) AND [2006-2016]/py \| 1711 \| 8 Aug 2016 \| \| #3 \| ('obesity'/exp OR 'childhood obesity'/exp OR 'body mass'/exp OR 'waist hip ratio'/exp OR 'overnutrition'/exp OR overweight*:ab,ti OR 'fat overload syndrome':ab,ti OR 'fat overload syndromes':ab,ti OR overeat*:ab,ti OR overfeed*:ab,ti OR adipos*:ab,ti OR obes*:ab,ti OR 'body mass index' OR bmi:ab,ti OR 'waist-hip ratio' OR 'skinfold thickness' OR 'abdominal fat') AND ('child care'/exp OR 'day care'/exp OR 'nursery school'/exp OR 'child care center':ab,ti OR 'nursery school':ab,ti OR 'nursery schools':ab,ti OR 'child care':ab,ti OR 'home-based day care':ab,ti OR 'family child care home':ab,ti OR 'family child care homes':ab,ti OR 'child care centers':ab,ti OR 'daycare':ab,ti) \| 2033 \| 8 Aug 2016 \| \| #2 \| 'child care'/exp OR 'day care'/exp OR 'nursery school'/exp OR 'child care center':ab,ti OR 'nursery school':ab,ti OR 'nursery schools':ab,ti OR 'child care':ab,ti OR 'home-based day care':ab,ti OR 'family child care home':ab,ti OR 'family child care homes':ab,ti OR 'child care centers':ab,ti OR 'daycare':ab,ti \| 68643 \| 8 Aug 2016 \| \| #1 \| 'obesity'/exp OR 'childhood obesity'/exp OR 'body mass'/exp OR 'waist hip ratio'/exp OR 'overnutrition'/exp OR overweight*:ab,ti OR 'fat overload syndrome':ab,ti OR 'fat overload syndromes':ab,ti OR overeat*:ab,ti OR overfeed*:ab,ti OR adipos*:ab,ti OR obes*:ab,ti OR 'body mass index' OR bmi:ab,ti OR 'waist-hip ratio' OR 'skinfold thickness' OR 'abdominal fat' \| 728631 \| 8 Aug 2016 \| |
| --- | --- | --- | --- | --- | --- | --- | --- | --- | --- | --- | --- | --- | --- | --- | --- | --- | --- | --- | --- | --- |

## **CINAHL**

| **Search ID#** | **Search Terms** | **Search Options** | **Last Run Via** | **Results** |
| --- | --- | --- | --- | --- |
| S4 | S1 AND S2 | Limiters - Published Date: 20060101-20161231  Search modes - Boolean/Phrase | Interface - EBSCOhost Research Databases  Search Screen - Advanced Search  Database - CINAHL Plus with Full Text | 327 |
| S3 | S1 AND S2 | Search modes - Boolean/Phrase | Interface - EBSCOhost Research Databases  Search Screen - Advanced Search  Database - CINAHL Plus with Full Text | 358 |
| S2 | ( (MH "Child Care") OR "child care" OR (MH "Child Day Care") OR (MH "Child Care Providers") OR (MH "Child Care (Saba CCC)") OR (MH "Day Care") OR (MH "Infant Care (Iowa NIC)") OR (MH "Schools, Nursery") ) OR ( "child care center" OR "nursery school" OR "nursery schools" OR "child care" OR "home-based day care" OR "family child care home" OR "family child care homes" OR "child care centers" OR "daycare" ) | Search modes - Boolean/Phrase | Interface - EBSCOhost Research Databases  Search Screen - Advanced Search  Database - CINAHL Plus with Full Text | 10,641 |
| S1 | ( (MH "Obesity+") OR "obesity" OR (MH "Attitude to Obesity+") OR (MH "Obesity, Morbid+") OR (MH "Adipose Tissue Distribution") OR "overweight" OR (MH "Body Mass Index") ) OR ( overweight* OR "fat overload syndrome" OR "fat overload syndromes" OR overeat* OR overfeed* OR adipos* OR obes* OR "body mass index" OR bmi OR "waist-hip ratio" OR "skinfold thickness" OR "abdominal fat" ) | Search modes - Boolean/Phrase | Interface - EBSCOhost Research Databases  Search Screen - Advanced Search  Database - CINAHL Plus with Full Text | 140,301 |

Searched 08082016

## PsycINFO

| **Search ID#** | **Search Terms** | **Search Options** | **Last Run Via** | **Results** |
| --- | --- | --- | --- | --- |
| S4 | S1 AND S2 | Limiters - Publication Year: 2006-2016  Search modes - Boolean/Phrase | Interface - EBSCOhost Research Databases  Search Screen - Advanced Search  Database - PsycINFO | 791 |
| S3 | S1 AND S2 | Search modes - Boolean/Phrase | Interface - EBSCOhost Research Databases  Search Screen - Advanced Search  Database - PsycINFO | 864 |
| S2 | (((DE "Obesity" OR DE "Obesity (Attitudes Toward)") OR (DE "Overweight")) OR (DE "Body Mass Index")) OR (DE "Body Fat") OR ( overweight* OR "fat overload syndrome" OR "fat overload syndromes" OR overeat* OR overfeed* OR adipos* OR obes* OR "body mass index" OR bmi OR "waist-hip ratio" OR "skinfold thickness" OR "abdominal fat" ) | Search modes - Boolean/Phrase | Interface - EBSCOhost Research Databases  Search Screen - Advanced Search  Database - PsycINFO | 48,877 |
| S1 | ((DE "Child Day Care" OR DE "Child Care" OR DE "Day Care Centers") AND (DE "Nursery School Students" OR DE "Nursery Schools")) OR (DE "Preschool Students") OR ( "child care center" OR "nursery school" OR "nursery schools" OR "child care" OR "home-based day care" OR "family child care home" OR "family child care homes" OR "child care centers" OR "daycare" ) | Search modes - Boolean/Phrase | Interface - EBSCOhost Research Databases  Search Screen - Advanced Search  Database - PsycINFO | 51,257 |

Searched 08082016
